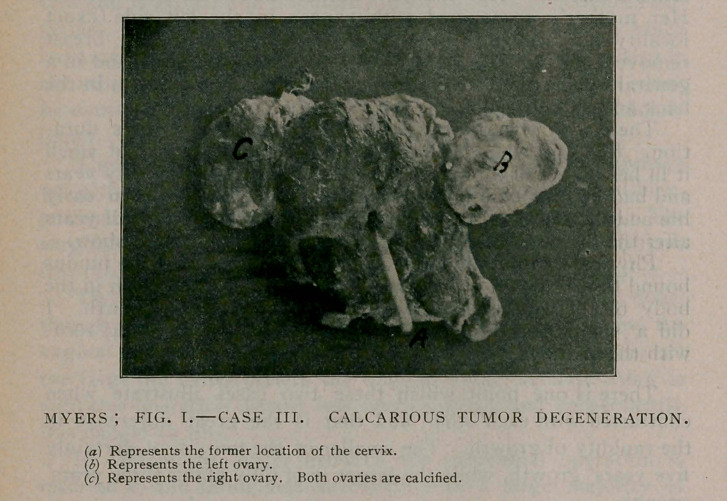# Uterine Fibroids or Myomata1Read at the 35th annual meeting of the Medical Association of Central New York, held at Syracuse, N. Y., October 21, 1902.

**Published:** 1903-02

**Authors:** J. F. Myers

**Affiliations:** Sodus, N. Y.


### Uterine Fibroids or Myomata.[1]

By J. F. MYERS, Sodus, N. Y.

A FIBROID or myoma is defined as a foreign growth or tumor composed of connective or muscular tissue.

Pathologists (Delafield and Pruden) divide myomata into two general classes in accordance with the physiological division of muscles in general: (1) cleiomyomata; (2) rhabdomyomata. We quote from these authors as follows:

In the first-class we find the characteristic elements to be fusiform smooth fibers with elongated rod-shaped nuclei. These fibers are packed closely together and may interlace, branching in different directions, and are intermingled with more or less vascular fibrillar connective tissue. When this

1. Read at the 35th annual meeting of the Medical Association of Central New York, held at Syracuse, N. Y., October 21, 1902.

connective tissue is present in large proportions the growth is called a fibromyoma. It is not always easy in sections to distinguish these myomata from certain cellular fibromata. But if we note the shape of isolated cells, their nuclei and their uniformity in size, it will usually suffice. These tumors sometimes become infiltrated with lime salts until they are completely calcified and stone-like. Because of their density they may undergo cystic degeneration, or a large portion of the tumor may become gangrenous.

The tumors of the fibrocystic form are the largest tumors which grow. Dr. Kelly describes a case in which the tumor weighed 59 pounds.

These tumors may be single or multiple, large or small, are of slow growth and benign. They occur most frequently in the uterus, where they may be multiple. They have been found wherever smooth muscular tissue exists. They may be found in the muscular coat of the gastrointestinal canal. They have been found in the bladder and the skin of the nipple and scrotum. The enlarged prostates of old men are cleiomyomata of the interstitial coat.

Class II.—In these rare tumors striated muscular fibers form the characteristic elements. They very rarely compose a great part of the tumor but are intermingled with other elements, fibrillar connective tissue, spindle-shaped and spheroidal cells of various forms, which often appear to be incompletely developed muscle cells. They are not infrequently associated with sarcomatous tissue. Bloodvessels and sometimes nerves are also present.

These muscle fibers differ as a rule from the normal striated muscle fibers in their arrangement, which is quite irregular, and in their size, being in general smaller than normal fibers although varying greatly. The sarcolemma is either absent or incompletely developed. These tumors are usually small or of moderate size and are supposed to originate from the inclusion of cells intended for muscle cells in places where they do not belong. In the heart and certain other muscular parts small circumscribed masses of striated muscle tissue have been described. They are sometimes called "homologous rhabdomyomata." But genuine heterologous rhabdomyomata are in almost all cases thus far recorded confined to the genitourinary organs, kidney, ovary and testicle.

Dr. Prudden describes a case of rhabdomyomata of the parotid gland, and says: "These tumors when not associated with sarcomatous tissue, are usually small and benign and are of more theoretical than practical importance."

When a woman in middle life consults the physician, complaining of prolonged, excessive and painful menstruation, with her periods extending over many days, so that she is sick the greater part of the month, being sterile, and perhaps having had one or more miscarriages in early life, the physician should suspect the presence of a myoma and advise a physical examination, which alone can decide its presence or absence. Putting the patient in the lithotomy position, with bimanual examination, we ascertain in a general way the size of the uterus, the presence and size of the tumor. Then, with the uterine sound, we ascertain the exact length of the uterine cavity. Then it is necessary to ascertain the relative position of the tumor to the womb.

Having first prepared the patient with an evening laxative and a morning enema, thoroughly emptying the lower bowel and rectum, with the patient in the lithotomy position, we seize the cervix with vollsellum and draw the uterus well down and may then place the forceps in the hands of an assistant. With one finger in the rectum and the other hand on the abdomen we can thoroughly examine any unevenness or nodules on the surface of the uterus and the relation of any foreign growth to it. We may often ascertain whether the myoma is pedunculate, sessile, interstitial, subserous or submucous, which knowledge is essential to a proper treatment.

### PROGNOSIS.

Comparatively few of these growths cause symptoms sufficient to be called to the attention of the surgeon, and the tumors themselves, unless so located as to cause symptoms, are histologically quite harmless. The submucous tumors, when occurring in young women, may, by causing menorrhagia, so weaken the patient as to incapacitate her and cause years of suffering. The menorrhagia may be so excessive as to endanger life at the menstrual period. Some growths originating in the cervix and extending up into the body of the uterus and tubes may cause so much pain as to render life a burden. Some of these submucous growths may be extruded from the cervix and can easily be removed as an ordinary polypus.

After the menopause myomata may atrophy, except where cystic degeneration has taken place. The fibrocystic tumors continue growing till by their size and weight they make life a burden. Myomata may by their position and form become lodged under the brim of the pelvis so that their pressure and growth is in the direction of least resistance on sensitive pelvic viscera, causing most uncomfortable symptoms. They may press upon the ureter, causing hydroureter, which may be followed by pyelitis and nephritis. They may undergo gangrenous degeneration and sepsis may follow, which is another menace to life. They may also be associated with sarcomatous tissue, which is sure to prove fatal if not removed early.

It thus becomes a serious matter to be the possessor of a myoma.

### TREATMENT.

Treatment is expectant or palliative, abortive and radical. The expectant or palliative treatment consists in watching and treating developing symptoms, which in some cases is to allow nature to take its course.

Menstrual hemorrhage, which Dr. Howard A. Kelly calls the most dreadful symptom of myomata and says occurs in 50 per cent. of cases, in the submucous varieties, may be treated by keeping the woman in bed during the menstrual period and administering ergot, opium and cannabis indica in proper doses.

Curetage of the endometrium with after applications of astringents is also an effective measure in these cases. Kelly also says that the most efficient alterative to the endometrium we have is the application of electricity. With the positive electrode in the cervix and the negative pole covering a large area on the abdomen we employ from 50 to 150 milliamperes.

In the abortive treatment small injections of ergot twice a day for two or three months has been known to cause a retrogression in their growth. A spear-pointed positive electrode, scrupulously aseptic, may be plunged into the substance of the submucous tumors and in a few cases will arrest their growth although accompanied with the dangers of sepsis and gangrenous degeneration. Ovariotomy was once a laudable abortive measure. Tait performed 271 ovariotomies to hasten the menopause and thus expedite their retrogression. He completely cured 257 cases. The writer does not often resort to the above forms of treatment because of danger of sepsis and gangrene.

In cases that lodge under the brim of the pelvis, if the adhesions are not too strong, we may attempt to lift them by the use of pessaries or tampons out of the pelvic into the abdominal cavity, where there is more room; or, they may be carefully manipulated where the adhesions and vascularity do not endanger life from internal hemorrhages and inflammations. In cases too difficult or dangerous for vaginal manipulation an exploratory laparatomy may be done; and where it is not feasible or possible to do a myomectomy or a hysterectomy, because of advanced age or an enfeebled condition of the system, the adhesions may be broken up to such an extent as to allow the myoma to be raised above the brim of the pelvis into the abdominal cavity, where it has more room. The writer never resorted to these palliative measures from choice, but solely where operation is refused and it is the only alternative.

Of the radical operations myomectomy is the more conservative and is growing in popularity in the hands of experienced surgeons. This is easily done in case of pedunculate tumors which have emerged from the interstitial tissue of the womb, and also in the subserous variety. Dr. Howard Kelly reports a case in which he enucleated nine fibromata, leaving the uterine tissue mostly intact.

These incisions must be very nicely closed and hemorrhage perfectly controlled. This operation is more dangerous in inexperienced hands because of post-operative hemorrhages. The surgical technique must be as nearly perfect as may be, to have this operation a success.

The hysteromyomectomy is more easily and frequently done, though less conservative. This can be done either by the vaginal or abdominal routes. In cases where the myoma is not too large the writer prefers the vaginal route, because there is with this operation usually less surgical shock and consequently less danger to life.

In case of the larger growths the abdominal operation is resorted to and terminates successfully in a large majority of cases. The technique of the above operations I will not attempt to describe as they are so familiar to all.

Case I.—Mrs. A., aged 45, entered the hospital October 20, 1901. There was a good family history, the parents and grand parents having lived to advanced ages. She had been suffering at irregular intervals with prolonged and excessive menstruation and constantly with pain, irritable bladder and other symptoms caused by pressure in such cases. She was quite anemic from the loss of blood at different times. Physical examination revealed the presence of the myoma you see here. I operated October 27, by laparatomy.

Her pulse was 150 before the operation. A hypodermic of strychnin and brandy brought it down nearly to the normal. I removed the uterus and one ovary. I had to rapidly bring the operation to a close because of symptoms of collapse. We administered hot salines and hypodermics of atropin and strychnin alternately, and by evening the patient came out from under the anesthetic with pulse of 140 to the minute. October 28 the pulse was 120 and temperature 99°.

October 29, the pulse was 100 and temperature 100°, after which time convalescence was uninterrupted.

Case II.—Mrs. B., age 39 years, entered the hospital November 30, 1901. Family history—her mother died two hours after her birth, the cause of the death being eclampsia. She was brought up by strangers and knew nothing of her father's history. Her mother's father died with tuberculosis. Her mother's sister died with cancer in the left side (exact locality not known.) One aunt had a cancer of the breast removed and is still living. She was very nervous and in a general hyperesthetic condition and complained of pain in the back and left side.

There was a history of severe obstipation of five years' duration. She said when her bowels did not move she could smell it in her nose and throat. She had been married twenty years and had had no children. Had had one miscarriage in early life and there was no menstrual flow for one and one-half years after the miscarriage, and always after that a very slight show.

Physical examination revealed a retroversion with the fundus bound tightly down on the rectum, and a small growth in the body of the womb which was at least of five years' growth. I did a vaginal hysteromyomectomy on her December 4, 1901, with the ordinary uninterrupted convalescence.

There is one point which these two cases illustrate when contrasted with each other, and that is the great difference in the rapidity of growth. The smaller of the two is of certainly five years' growth, while the larger is of only about three years' growth.

Case III.—Mrs. C., aged 62 years. Had been married 45 years. She was the mother of three children, all healthy. She entered the hospital December 3, complaining of darting pains beginning in the pelvis low down and shooting in all directions. She was also suffering from bronchitis and a very troublesome cough. She had stopped menstruating ten years previously. Physical examination revealed the presence of a uterine growth, and also acute bronchitis. After a week's treatment the bronchial trouble subsided.

Under protest, because of her age, I had her anesthetised and attempted a vaginal hysterectomy, but everything was so rigid about the pelvic organs it could not be done. I therefore did an abdominal hysteromyomectomy and removed this stone-like uterus, which I show you.

The ovaries were also calcified. The woman died in the evening. I afterward ascertained that she had impaired her nervous system by taking morphine and chloral hydrate. Had I known this, I should not have attempted the operation under any consideration.

This uterus, as you see, has undergone a calcareous degeneration and is perfectly stone-like. Of course, this would have remained dormant and never have grown any, but in this particular case caused sharp, darting, burning pains, radiating in all directions, for which cause it was removed.

MYERS; FIG. I.—CASE III. CALCARIOUS TUMOR DEGENERATION.

(*a*) Represents the former location of the cervix.
(*b*) Represents the left ovary.
(*c*) Represents the right ovary. Both ovaries are calcified.

Case IV.—Mrs. J. D., aged 27 years, entered hospital complaining of a very troublesome muco-sanguineous discharge lasting from one menstrual period to the next. This discharge was nearly constant for three years. Her father and mother, brothers and sisters all living and in good health. Her husband was in good health. Physical examination revealed a cauliflower growth in the cervix which was much larger in life than the specimen is now. I did a vaginal hysteromyomectomy on her after the usual preparation. She was up and dressed in two weeks and was discharged from the hospital three weeks after the operation.

Case V.—Mrs. E., aged 30, came into the hospital June 25, 1902, complaining of pelvic pains and distress of five years' standing. Her family history was good. The father and mother were alive and in good health. She complained of pain in the back and an irritable bladder and dyspareunia which seemed to plague her more than anything else. Physical examination revealed a uterus in the second degree of prolapse bound down firmly. There was also subinvolution. The womb was large and very sensitive to the touch. There was an endometritis.

She was told that she could have a curetment done and adhesions loosened and round ligaments shortened, or a hysterectomy. She decided to have the hysterectomy done. I therefore did a vaginal hysterectomy on June 30. The patient was up and dressed July 10, and discharged from hospital July 19, convalescence being uninterrupted.

## Figures and Tables

**Fig. I. f1:**